# Association of pathway mutation with survival after recurrence in colorectal cancer patients treated with adjuvant fluoropyrimidine and oxaliplatin chemotherapy

**DOI:** 10.1186/s12885-019-5650-0

**Published:** 2019-05-06

**Authors:** Dae-Won Lee, Sae-Won Han, Yongjun Cha, Jeong Mo Bae, Hwang-Phill Kim, Jaemyun Lyu, Hyojun Han, Hyoki Kim, Hoon Jang, Duhee Bang, Jae-Kyung Won, Seung-Yong Jeong, Kyu Joo Park, Gyeong Hoon Kang, Tae-You Kim

**Affiliations:** 1 Department of Internal Medicine, Seoul National University Hospital, 101 Daehang-Ro, Jongno-Gu, Seoul, 110-744 South Korea; 2 Cancer Research Institute, Seoul National University College of Medicine, Seoul, South Korea; 3 Department of Pathology, Seoul National University College of Medicine, Seoul, South Korea; 4 Department of Molecular Medicine & Biopharmaceutical Sciences, Graduate School of Convergence Science and Technology, Seoul National University, Seoul, South Korea; 5 Celemics Inc, Seoul, South Korea; 6 Department of Chemistry College of Science, Yonsei University, Seoul, South Korea; 7 Department of Surgery, Seoul National University Hospital, Seoul, South Korea

**Keywords:** Colorectal cancer, TGF-β pathway, Mucinous adenocarcinoma, Survival after recurrence

## Abstract

**Background:**

Although the prognostic biomarkers associated with colorectal cancer (CRC) survival are well known, there are limited data on the association between the molecular characteristics and survival after recurrence (SAR). The purpose of this study was to assess the association between pathway mutations and SAR.

**Methods:**

Of the 516 patients with stage III or high risk stage II CRC patients treated with surgery and adjuvant chemotherapy, 87 who had distant recurrence were included in the present study. We analyzed the association between SAR and mutations of 40 genes included in the five critical pathways of CRC (WNT, P53, RTK-RAS, TGF-β, and PI3K).

**Results:**

Mutation of genes within the WNT, P53, RTK-RAS, TGF-β, and PI3K pathways were shown in 69(79.3%), 60(69.0%), 57(65.5%), 21(24.1%), and 19(21.8%) patients, respectively. Patients with TGF-β pathway mutation were younger and had higher incidence of mucinous adenocarcinoma (MAC) histology and microsatellite instability-high. TGF-β pathway mutation (median SAR of 21.6 vs. 44.4 months, *p* = 0.021) and MAC (20.0 vs. 44.4 months, *p* = 0.003) were associated with poor SAR, and receiving curative resection after recurrence was associated with favorable SAR (Not reached vs. 23.6 months, *p* <  0.001). Mutations in genes within other critical pathways were not associated with SAR. When MAC was excluded as a covariate, multivariate analysis revealed TGF-β pathway mutation and curative resection after distant recurrence as an independent prognostic factor for SAR. The impact of TGF-β pathway mutations were predicted using the PROVEAN, SIFT, and PolyPhen-2. Among 25 mutations, 23(92.0%)-24(96.0%) mutations were predicted to be damaging mutation.

**Conclusions:**

Mutation in genes within TGF-β pathway may have negative prognostic role for SAR in CRC. Other pathway mutations were not associated with SAR.

**Electronic supplementary material:**

The online version of this article (10.1186/s12885-019-5650-0) contains supplementary material, which is available to authorized users.

## Background

Colorectal cancer (CRC) is ranked third in cancer incidence and second in cancer-related mortality worldwide [1]. CRC is a heterogeneous disease, and recent data from gene expression profiling have classified CRC into four consensus molecular subtypes (CMSs) [2]. In addition, integrated analysis of somatic mutations, copy number change, and mRNA expression from the Cancer Genome Atlas (TCGA) revealed that the WNT, P53, RTK-RAS, TGF-β, and PI3K pathways are frequently altered in CRC [3, 4].

The current standard care in stage III CRC is complete resection of the tumor followed by adjuvant chemotherapy with 5-fluorouracil, leucovorin, and oxaliplatin (FOLFOX) or capecitabine plus oxaliplatin (XELOX) [5, 6]. However, approximately 30% of patients develop recurrence despite receiving adjuvant chemotherapy [7]. Several biomarkers, including microsatellite instability-high (MSI-H), the CpG island methylator phenotype (CIMP), *KRAS* mutation, and *BRAF* mutation, are associated with recurrence and survival in CRC patients [8–11]. Although the prognostic biomarkers associated with cancer recurrence are well studied, there is a paucity of data regarding biomarkers associated with survival after recurrence (SAR). Recently, from a study population of stage III CRC patients enrolled in a phase III adjuvant chemotherapy study, MSI-H was associated with improved SAR, and *KRAS* mutation and *BRAF* mutation were associated with poor SAR [12].

We have previously reported that mutation in genes within PI3K pathway is associated with better RFS and that mutation in genes within RTK-RAS pathway is associated with worse RFS in CRC treated with adjuvant chemotherapy [13]. The purpose of this study was to evaluate the association between five critical pathway mutations and SAR in CRC patients who had distant recurrence after the treatment of curative surgery followed by adjuvant fluoropyrimidine and oxaliplatin chemotherapy.

## Methods

### Patients and treatment

The study population consisted of patients with stage III or high-risk stage II CRC who developed distant recurrence after receiving curative surgery at Seoul National University Hospital (SNUH, Seoul, South Korea). All patients were included in the CRC patient cohort reported previously [13]. Patients received at least 6 cycles of adjuvant FOLFOX or 4 cycles of adjuvant XELOX chemotherapy. Upper rectal cancer patients were included if they did not receive pre- or post-operative radiation therapy. Exclusion criteria were the following: signet ring cell histology, anti-*EGFR* or anti-*VEGF* treatment for adjuvant chemotherapy, history of other malignancy within 5 years, and local recurrence only. Adjuvant FOLFOX was planned for 12 cycles and XELOX chemotherapy was planned for 8 cycles. 50 patients received FOLFOX-4, 27 received FOLFOX-6, and 10 received XELOX. Electronic database and electronic medical record system of SNUH was used to identify eligible patients and review their medical charts.

### Molecular testing, including targeted sequencing of 40 genes associated with five critical pathways

Every exon of the 40 genes associated with the five critical pathways of CRC was sequenced [13]. Fourteen genes were selected from WNT pathway (*ARID1A, AMER1, APC, AXIN2, CTNNB1, DKK1, DKK2, DKK3, DKK4, FBXW7, FZD10, LRP5, SOX9, TCF7L2*), 2 genes from P53 pathway (*ATM, TP53*), 8 genes from RTK-RAS pathway (*BRAF, EGFR, ERBB2, ERBB3, ERBB4, HRAS, KRAS, NRAS*), 7 genes from TGF-β pathway (*ACVR1B, ACVR2A, SMAD2, SMAD3, SMAD4, TGFBR1, TGFBR2*), and 9 genes from PI3K pathway (*IGF1R, IGF2, IRS2, MTOR, PDGFRA, PIK3CA, PIK3R1, PTEN, SRC*). Detailed methods for targeted sequencing can be found in our previous article [13]. Briefly, genomic DNA (> 200 ng) samples were sheared and prepared according to routine library preparation. The captured library was amplified and sequenced using Hiseq 2500 (Illumina, USA). Sequencing data were filtered with a mean quality Q20 (Phred score) per read, and these filtered data were aligned to GRCh37 using bwa 0.7.5a. The aligned reads were processed with Picard Mark Duplicates and GATK base recalibration. After a series of processes, the aligned bases were piled up with SAM tools. Variant call and somatic analysis processes were performed by Varscan and were annotated with ANNOVAR. The pathways were defined as having a mutation if any gene included in the pathway had a mutation.

The microsatellite status was assessed using the following markers: BAT25, BAT26, D2S123, D5S346 and D17S250 [13]. Instability at 2 or more markers were defined as MSI-high, 1 as MSI-low, and 0 as MSS. MSI-H was regarded as having MSI, and MSI-L was grouped with MSS.

### Statistical analysis

The primary objective of this study was to investigate the effect of mutations of critical pathways and clinico-pathologic characteristics on SAR. SAR was defined as the time from distant tumor recurrence to death from any cause. Categorical variables were compared by chi-square test or Fisher’s exact test. Continuous variables were compared using the independent-samples T test. SAR was calculated using the Kaplan-Meier method, and comparisons were made using log-rank tests. Hazard ratios (HRs) were calculated using the Cox proportional hazard model. To adjust for the baseline characteristics, Cox proportional hazard analysis of SAR included sex, age (continuous variable), tumor location [proximal (from the cecum to the transverse colon) vs. distal (from the descending colon to the rectum)], tumor histology (mucinous adenocarcinoma vs. non-mucinous adenocarcinoma), tumor (T) stage (continuous variable), lymph node (N) stage (continuous variable), curative operation (done vs. not done), recurrence before 1 year, microsatellite status (MSI-high vs. MSS and MSI-low), and five critical pathway mutations. Two-sided *p*-values less than 0.05 were considered statistically significant. Statistical analysis was performed using SPSS software for Windows, version 20.0 (SPSS, Chicago, IL, USA).

## Results

### Patient characteristics

At a median follow-up duration of 44.4 months, 87 patients had distant recurrence among our database of 516 patients [13]. The baseline characteristics of the 87 patients are summarized in Table 1. The primary tumor location was proximal (from the cecum to the transverse colon) in 28 patients (32.2%) and distal (from the descending colon to the rectum) in 59 patients (67.8%). Nine patients (10.3%) had MAC histology, and 3 patients (3.5%) showed MSI-H feature.

**Table 1:** Baseline characteristics

Characteristics		Total (*N* = 87)	TGF-β WT (*N* = 66)	TGF-β MT (*N* = 21)	*P*-Value
Age	Median (range)	59 (31–75)	61 (38–75)	54 (31–74)	0.020
Sex	Male	50 (57.5%)	36 (54.4%)	14 (66.7%)	0.33
	Female	37 (42.5%)	30 (45.5%)	7 (33.3%)	
Location	Proximal	28 (32.2%)	19 (28.8%)	9 (42.9%)	0.23
	Distal	59 (67.8%)	47 (71.2%)	12 (57.1%)	
Stage	II, high-risk	14 (16.1%)	8 (12.1%)	6 (28.6%)	0.074
	III	73 (83.9%)	58 (87.9%)	15 (71.4%)	
T stage	1	0 (0.0%)	0 (0.0%)	0 (0.0%)	0.41
	2	1 (1.1%)	1 (1.5%)	0 (0.0%)	
	3	62 (71.3%)	49 (74.2%)	13 (61.9%)	
	4	24 (27.6%)	16 (24.2%)	8 (38.1%)	
N stage	0	14 (16.1%)	8 (12.1%)	6 (28.6%)	0.18
	1	29 (33.3%)	24 (36.4%)	5 (23.8%)	
	2	44 (50.6%)	34 (51.5%)	10 (47.6%)	
Histology	Non-MAC	78 (89.7%)	65 (98.5%)	13 (61.9%)	< 0.001
	MAC	9 (10.3%)	1 (1.5%)	8 (38.1%)	
MSI	MSS/MSI-L	83 (96.5%)	65 (100.0%)	18 (85.7%)	0.013
	MSI-H	3 (3.5%)	0 (0.0%)	3 (14.3%)	
Time to recurrence	< 1 year	19 (21.8%)	11 (16.7%)	8 (38.1%)	0.038
	≥1 year	68 (78.2%)	55 (83.3%)	13 (61.9%)	
Curative resection after distant recurrence	Not done	52 (59.8%)	39 (59.1%)	13 (61.9%)	0.82
	Done	35 (40.2%)	27 (40.9%)	8 (38.1%)	

Abbreviations: *MAC* mucinous adenocarcinoma, *MSS* microsatellite stable, *MSI-L* microsatellite instability-low, *MSI-H* microsatellite instability-high

After distant recurrence, palliative chemotherapy was introduced in 77 (88.5%) patients. The first-line chemotherapy regimens included FOLFIRI in 56 patients, capecitabine monotherapy in 8, FOLFIRI plus bevacizumab in 7, FOLFIRI plus cetuximab in 2, FOLFOX in 2, S-1 plus oxaliplatin in 1, and capecitabine plus irinotecan in 1. Second-line chemotherapy was administered to 47 (54.0%) patients. Only 1 patient received both bevacizumab and cetuximab during the course of treatment. In total, 9 (10.3%) patients received bevacizumab and 9 (10.3%) patients received cetuximab during their course of treatment.

### Gene mutation and pathway mutation

In patients with recurrence, mutations were most frequently found in *TP53* (65.5%) followed by *APC* (64.4%), *KRAS* (48.3%), *FBXW7* (16.1%), and *SMAD4* (10.3%) (Additional file 1: Table S1). The WNT pathway was mutated in 79.3%, P53 in 69.0%, RTK-RAS in 65.5%, TGF-β in 24.1%, and PI3K in 21.8% of patients. The mutation frequencies of most genes were similar between the patients with recurrence and without recurrence (Additional file 1: Table S1). The mutation frequency of *PIK3CA* and *CTNNB1* was lower in the patients with recurrence, and *APC* showed a tendency of lower frequency in patients with recurrence. PI3K pathway mutation showed a tendency of lower frequency in patients with recurrence.

Patients with mutation in genes within TGF-β pathway showed distinct characteristics compared with patients without TGF-β pathway mutation (Table 1). Patients with TGF-β pathway mutation was associated with a younger age (median age of 54 years vs. 61 years, *p* = 0.020), and there was a tendency of a higher percentage of patients with initial high-risk stage II disease (28.6% vs. 12.1%, *p* = 0.074). Moreover, patients with TGF-β pathway mutation had a higher incidence of MSI-H and MAC. All 3 patients with recurrence in MSI-H had TGF-β pathway mutation. In addition, the incidence of TGF-β pathway mutation was 88.9% (8/9) in MAC patients compared with 16.7% (13/78) in non-MAC patients (*p* <  0.001) (Additional file 1: Table S2). The mutation incidences of other pathways were similar between MAC and non-MAC histology (Additional file 1: Table S2).

### Prognostic role of five critical pathways

**Fig. 1:**
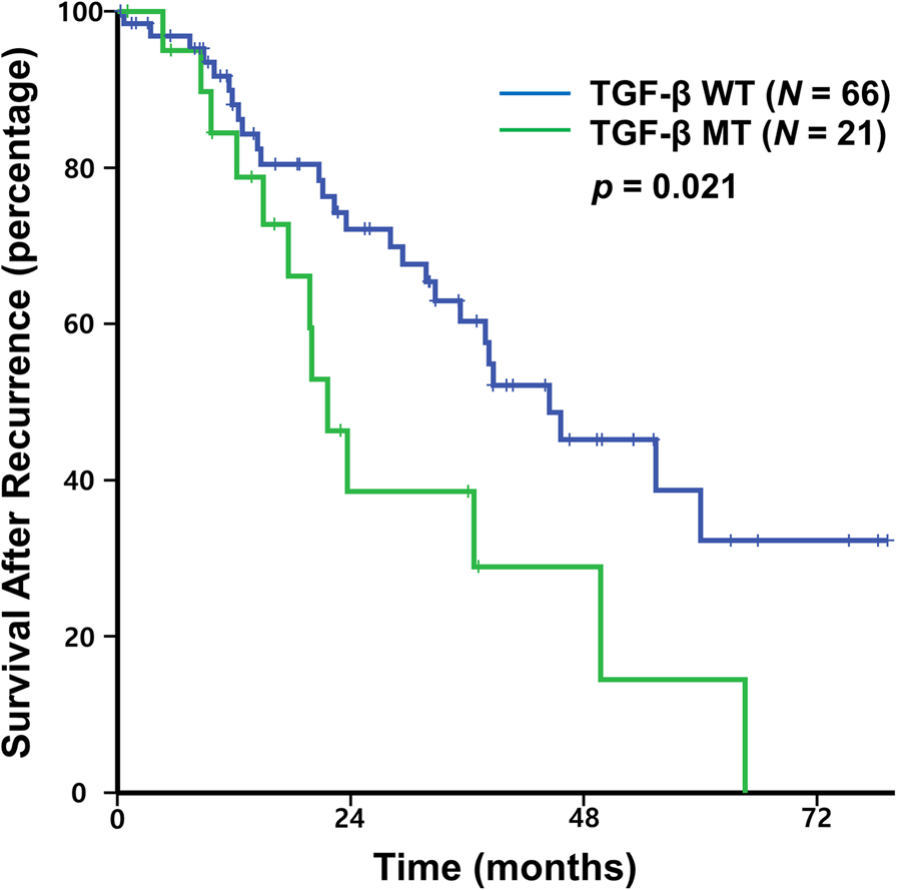
TGF-β pathway mutation and survival after recurrence

**Fig. 2:**
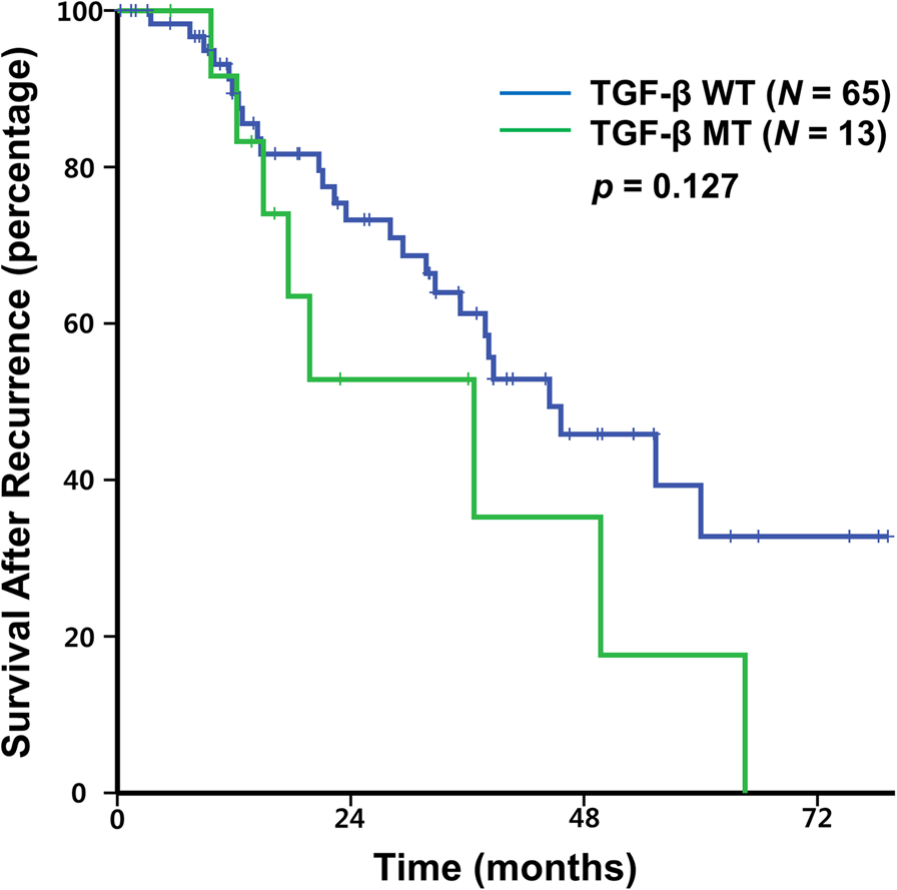
TGF-β pathway mutation and survival after recurrence in Non-MAC patients. Abbreviations: MAC, mucinous adenocarcinoma

Among the 87 patients who had distant recurrence, 40 death events occurred with an estimated median SAR of 38.2 months. SAR was significantly worse in patients with TGF-β pathway mutation than in those with wild type (median SAR of 21.6 vs. 44.4 months, *p =* 0.021) (Fig. 1). In our previous report, mutation in genes within PI3K pathway was associated with a longer RFS, whereas mutation in genes within RTK-RAS pathway was associated with a shorter RFS [13]. However, there was no correlation between SAR and other critical pathway mutations (Additional file 2: Figure S1). In addition to TGF-β pathway mutation, curative resection after distant recurrence and MAC was associated with poor SAR, and N2 stage showed a tendency of poor SAR (Table 2). Tumor location and MSI were not associated with SAR. Multivariate analysis using the Cox proportional hazard model revealed that curative resection after distant recurrence [adj HR for SAR 0.27 (95% CI 0.13–0.56), *p* = 0.001] and MAC histology [adj HR for SAR 3.77 (95% CI 1.52–9.35), *p* = 0.004] were independent prognostic factors for SAR. In the present study, MAC histology showed a higher incidence of TGF-β pathway mutation compared to non-MAC histology (88.9% vs. 16.7%, *p* <  0.001) (Additional file 1: Table S2). Although statistically not significant, the negative prognostic role of TGF-β pathway mutation was maintained in patients with non-MAC histology (median SAR of 36.7 vs. 44.4 months, *p =* 0.127) (Fig. 2). After removing MAC histology as a covariate, multivariate analysis revealed TGF-β pathway mutation [adj HR for SAR 1.99 (95% CI 1.02–3.90), *p* = 0.044] and curative resection after distant recurrence [adj HR for SAR 0.28 (95% CI 0.13–0.59), *p* = 0.001] as independent prognostic factors for SAR.

**Table 2:** Univariate analysis of survival after recurrence

Variable		Median SAR (months)	*p*-value
Age	< 65 years	36.7 (26.0–47.3)	0.71
	≥65 years	45.6 (29.4–61.8)	
Sex	Male	37.8 (33.6–42.1)	0.49
	Female	55.4 (0.0–110.8)	
Location	Proximal	37.8 (13.9–61.8)	0.82
	Distal	38.7 (27.9–49.4)	
Time to recurrence	< 1 year	31.7 (5.9–57.6)	0.16
	≥1 year	44.4 (31.4–57.4)	
Stage	II, high risk	49.7 (N/A)	0.36
	III	37.8 (30.2–45.5)	
T-stage	T1–3	38.2 (29.0–47.4)	0.31
	T4	28.1 (0.0–64.8)	
N-stage	N0–1	49.7 (26.1–73.3)	0.064
	N2	29.3 (11.4–47.3)	
Histology	Non-MAC	44.4 (31.2–54.7)	0.003
	MAC	20.0 (4.9–35.0)	
Curative resection after distant recurrence	Not done	23.6 (12.1–35.2)	< 0.001
	Done	Not reached	
MSI	MSS/MSI-L	38.2 (28.2–48.2)	0.35
	MSI-H	9.3 (1.7–38.2)	

Abbreviations: *MAC* mucinous adenocarcinoma, *MSS* microsatellite stable, *MSI-L* microsatellite instability-low, *MSI-H* microsatellite instability-high, *N/A* not available

We next analyzed whether individual gene mutation is associated with SAR (Additional file 1: Table S2). Patients with *SMAD4* mutation had worse SAR compared to wild type (SAR of 17.6 months vs. 38.7 months, *p* = 0031) and those with *APC* mutation had a tendency of favorable SAR compared to wild type (SAR of 45.6 months vs. 28.1 months, *p* = 0070). In the multivariate analysis, *SMAD4* mutation was an independent negative prognostic factor for SAR [adjusted hazard ratio (adj HR) for SAR 3.54 (95% CI 1.13–11.03), *p* = 0.030]. The detailed mutation profile of TGF-β pathway is shown in Table S4 (Additional file 1: Table S4). Among 25 mutations, 5 were stop gain mutation, 4 were frame shift deletion mutation, and 2 were non-frameshift deletion mutation which would probably lead to loss of expression. Impact of 14 non-synonymous SNV in TGF-β pathway mutation was predicted using the PROVEAN, SIFT, and PolyPhen-2 (Additional file 1: Table S2) [14–16]. Among 14 non-synonymous SNV, 12(85.7%)-13(92.9%) were predicted to be damaging mutation (Additional file 1: Table S4). In total, 23(92.0%)-24(96.0%) mutations were predicted to be damaging mutation in the TGF-β pathway. We also predicted functional outcome of *SMAD4* mutations using the OncoKB database (Additional file 1: Table S4) [17]. Among 9 *SMAD4* mutations, 3 mutations were found to be loss-of-function and additional 2 mutations involved the same location with aforementioned loss-of-function mutation. *ACVR1B* and *ACVR2A* genes were not covered by the OncoKB and our mutation involving *SMAD2*, *SMAD3*, *TGFBR1*, and *TGFBR2* was not reported in the OncoKB.

## Discussion

This study revealed that mutation in genes within TGF-β pathway is associated with MAC and may have poor SAR in colorectal cancer patients. Other critical pathways were not associated with SAR. In our previous study, mutation in genes within TGF-β pathway was not associated with RFS [13]. This implicates that while TGF-β pathway mutation is not associated with recurrence in early-stage tumors, it may be fatal in later-stage tumors.

In the present study, patients with TGF-β pathway mutation had higher incidence of MAC. This is in line with data from the TCGA where TGF-β pathway is more frequently altered in MAC histology compared to non-MAC histology (67.9% vs. 30.6%, *p* <  0.001) [18]. The TGF-β pathway is associated with cell proliferation, cell differentiation, apoptosis, and epithelial-mesenchymal transition (EMT) [19, 20]. Although mutational inactivation of TGF- β pathway is frequently shown in colorectal cancer, the level of TGF-b production is somehow elevated in later stage tumor [21, 22]. One of the reasons is that TGF-b level is not only affected by tumor but is also affected by stromal cells [22, 23]. The correlation between TGF- β pathway mutation (which is usually loss-of function) and TGF-b level has not been clearly identified and maybe different among disease status. In terms of clinical relevance, there are reports showing that TGF-β pathway may have a prognostic role in CRC. CMS classifies colorectal cancer based on the gene expression profile [2]. CMS4, which is characterized by the upregulation of genes implicated in EMT, prominent TGF-β pathway activation, stromal invasion, and angiogenesis, is associated with a worse prognosis. In addition, preclinical studies show that activation of TGF-β pathway may be linked to chemotherapy resistance in CRC cell line [24, 25]. Recombinant TGF-β treatment caused chemotherapy resistance in CRC cell lines [24]. However, TGF-β pathway mutation was not associated with RFS in CRC patients treated with adjuvant FOLFOX/XELOX chemotherapy, which we have previously reported [13]. Discordance in the prognostic role of TGF-β pathway mutation among disease status could be partially explained by the fact that the response to TGF-β pathway activation is different according to cell type and status [20, 26]. There are opposite effects of the TGF-β pathway in that it may suppress the growth of tubular adenoma but may promote the tumor growth of sessile serrated adenomas by inducing EMT and metastasis [20, 26]. Apoptosis is the dominant feature after TGF-β pathway stimulation in classical tubular adenoma organoid culture [26]. By contrast, inducing EMT is the main outcome after TGF-β pathway stimulation in an organoid culture model for sessile serrated adenomas [26]. The activation of TGF-β may suppress the growth of tubular adenoma but may progress sessile serrated adenomas to CMS4 subtype cancer by inducing EMT [26]. Moreover, evidence show that TGF-b level is also affected by stromal cells [22]. While TGF-β pathway mutation is not associated with RFS in CRC patients treated with adjuvant FOLFOX/XELOX chemotherapy, it may be fatal in CRC patients who have recurred after curative surgery. Among the genes involved in the TGF-β pathway, *SMAD4* mutation was associated with poor SAR. *SMAD4* is a tumor suppressor gene and its mutation is involved in advanced stages, such as distant metastasis, in human colorectal carcinogenesis [27]. In the present study, we predicted outcome of TGF-β pathway mutation using the PROVEAN, SIFT, and PolyPhen-2. Among 14 non-synonymous SNV in TGF-β pathway mutation 12(85.7%)-13(92.9%) were predicted to be damaging mutation. Other 11 mutations consisted of 5 stop gain mutation, 4 frame shift deletion mutation, and 2 non-frameshift deletion mutation which would probably lead to loss of expression. In sum, 23(92.0%)-24(96.0%) mutations were predicted to be damaging mutation in the TGF-β pathway. In addition, all SMAD4 mutations reported in the OncoKB database were loss-of-function mutation. This shows that TGF-β pathway mutation may have contributed to poor SAR due to loss of function. The correlation between TGF-β pathway mutation, TGF-b expression, and chemotherapy resistance needs to be studied in the future.

Sinicrope et al. reported that MSI-H is associated with improved SAR and that *KRAS* mutation and *BRAF* mutation are associated with poor SAR in stage III colon cancer patients treated with adjuvant chemotherapy [12]. However, we could not evaluate the role of MSI-H and *BRAF* mutation in SAR because there were only 3 patients with MSI-H and 5 patients with *BRAF* V600E mutation. In contrast to a study by Sinicrope et al., RTK-RAS pathway mutation and *KRAS* exon 2 mutation were not associated with SAR in the present study. In a study by Sinicrope et al., the negative prognostic role of *KRAS* mutation was limited to patients who received adjuvant FOLFOX pus cetuximab but not in patients treated with adjuvant FOLFOX alone. In the present study, none of the patients received cetuximab in the adjuvant setting. This implicates that *KRAS* mutation may not affect SAR in patients treated with adjuvant FOLFOX or XELOX alone.

The major limitation of this study was that we did not evaluate genetic characteristics other than targeted sequencing of genes associated with 5 critical pathways. Although major genes included in the TGF-β pathway were sequenced, other alterations, including copy number variation or the expression level, were not analyzed. It is also important to analyze the TGF-β pathway induced by stromal cells [3, 23]. Therefore, comprehensive analysis of the TGF-β pathway could provide a better understanding of CRC biology in the future. Another limitation of the present study was that patients were treated differently after recurrence. However, this study was performed at a high-volume single center where the treatment plan was relatively uniform. The last limitation was the relatively small number of patients included in this cohort. However, this was the first study to evaluate the relationship between pathway mutation and SAR in CRC patients.

## Conclusions

This study revealed that CRC patients with mutation in genes within TGF-β pathway may have poor SAR. Other pathway mutations were not associated with SAR. Future works on the relationship between TGF-β pathway mutation and survival according to disease status needs to be studied.

## Additional files

Additional file 1: **Table S1.** Mutation rate of 40 genes included in the study. **Table S2.** Mutation rate of critical pathways according to histology. **Table S3.** Survival after recurrence according to each gene mutation. (Genes with mutation rate over 5%). **Table S4.** Detailed profile of TGF-β pathway mutation. (DOCX 60 kb)

Additional file 2: **Figure S1**. Pathway mutations and survival after recurrence. Mutation in pathways other than TGF-β were not associated with survival after recurrence. (TIF 311 kb)

## Abbreviations

CI: Confidence interval
CIMP: CpG island methylator phenotype
CMS: Consensus molecular subtypes
CRC: Colorectal cancer
EMT: Epithelial-mesenchymal transition
FOLFOX: 5-fluorouracil leucovorin, and oxaliplatin
HR: Hazard ratio
MAC: Mucinous adenocarcinoma
MSI: Microsatellite instability
MSS: Microsatellite-stable
OS: Overall survival
RFS: Relapse-free survival
SAR: Survival after recurrence
SNUH: Seoul National University Hospital
TCGA: The Cancer Genome Atlas
XELOX: Capecitabine plus oxaliplatin
