# Supplementary material for: Association of pathway mutation with survival after recurrence in colorectal cancer patients treated with adjuvant fluoropyrimidine and oxaliplatin chemotherapy
Source: BMC Cancer. 2019 May 6;19:421. doi: 10.1186/s12885-019-5650-0 (PMC6501409; doi:10.1186/s12885-019-5650-0)
Supplement: Supplementary file 1 — Table S1. Mutation rate of 40 genes included in the study. Table S2. Mutation rate of critical pathways according to histology. Table S3. Survival after recurrence according to each gene mutation. (Genes with mutation rate over 5%). Table S4. Detailed profile of TGF-β pathway mutation. (DOCX 60 kb) [file 12885_2019_5650_MOESM1_ESM.docx]

**Table S1. Mutation rate of 40 genes included in the study**

| Gene | Total  (*N* = 516) | Not-recurred (*N* = 429) | Recurred (*N* = 87) | *P-*Value |
| --- | --- | --- | --- | --- |
| WNT Pathway | 436 (84.5%) | 367 (85.5%) | 69 (79.3%) | 0.14 |
| *AMER1* | 51 (9.9%) | 43 (10.0%) | 8 (9.2%) | 0.81 |
| *APC* | 373 (72.3%) | 317 (73.9%) | 56 (64.4%) | 0.070 |
| *ARID1A* | 56 (10.9%) | 49 (11.4%) | 7 (8.0%) | 0.36 |
| *AXIN2* | 31 (6.0%) | 26 (6.1%) | 5 (5.7%) | 0.91 |
| *CTNNB1* | 31 (6.0%) | 30 (7.0%) | 1 (1.1%) | 0.044 |
| *DKK1* | 5 (1.0%) | 5 (1.2%) | 0 (0.0%) | 0.60 |
| *DKK2* | 4 (0.8%) | 4 (0.9%) | 0 (0.0%) | 1.00 |
| *DKK3* | 3 (0.6%) | 3 (0.7%) | 0 (0.0%) | 1.00 |
| *DKK4* | 7 (1.4%) | 6 (1.4%) | 1 (1.1%) | 1.00 |
| *FBXW7* | 98 (19.0%) | 84 (19.6%) | 14 (16.1%) | 0.45 |
| *FZD10* | 18 (3.5%) | 16 (3.7%) | 2 (2.3%) | 0.75 |
| *LRP5* | 20 (3.9%) | 17 (4.0%) | 3 (3.4%) | 1.00 |
| *SOX9* | 50 (9.7%) | 44 (10.3%) | 6 (6.9%) | 0.33 |
| *TCF7L2* | 66 (12.8%) | 60 (14.0%) | 6 (6.9%) | 0.071 |
| P53 pathway | 356 (69.0%) | 296 (69.0%) | 60 (69.0%) | 1.00 |
| *ATM* | 49 (9.5%) | 44 (10.3%) | 5 (5.7%) | 0.19 |
| *TP53* | 329 (63.8%) | 272 (63.4%) | 57 (65.5%) | 0.71 |
| RTK-RAS pathway | 313 (60.7%) | 256 (59.7%) | 57 (65.5%) | 0.31 |
| *BRAF* | 47 (9.1%) | 39 (9.1%) | 8 (9.2%) | 0.98 |
| *EGFR* | 20 (3.9%) | 18 (4.2%) | 2 (2.3%) | 0.55 |
| *ERBB2* | 39 (7.6%) | 34 (7.9%) | 5 (5.7%) | 0.48 |
| *ERBB3* | 31 (6.0%) | 27 (6.3%) | 4 (4.6%) | 0.80 |
| *ERBB4* | 30 (5.8%) | 27 (6.3%) | 3 (3.4%) | 0.45 |
| *HRAS* | 2 (0.4%) | 2 (0.5%) | 0 (0.0%) | 1.00 |
| *KRAS* | 223 (43.2%) | 181 (42.2%) | 42 (48.3%) | 0.30 |
| *NRAS* | 19 (3.7%) | 16 (3.7%) | 3 (3.4%) | 1.00 |
| PI3K pathway | 155 (30.0%) | 136 (31.7%) | 19 (21.8%) | 0.067 |
| *IGF1R* | 15 (2.9%) | 14 (3.3%) | 1 (1.1%) | 0.49 |
| *INS-IGF2* | 13 (2.5%) | 9 (2.1%) | 4 (4.6%) | 0.25 |
| *IRS2* | 14 (2.7%) | 14 (3.3%) | 0 (0.0%) | 0.14 |
| *MTOR* | 30 (5.8%) | 28 (6.5%) | 2 (2.3%) | 0.20 |
| *PDGFRA* | 17 (3.3%) | 16 (3.7%) | 1 (1.1%) | 0.33 |
| *PIK3CA* | 91 (17.6%) | 83 (19.3%) | 8 (9.2%) | 0.023 |
| *PIK3R1* | 19 (3.7%) | 17 (4.0%) | 2 (2.3%) | 0.75 |
| *PTEN* | 21 (4.1%) | 20 (4.7%) | 1 (1.1%) | 0.23 |
| *SRC* | 5 (1.0%) | 3 (0.7%) | 2 (2.3%) | 0.20 |
| TGF-β pathway | 149 (28.9%) | 128 (29.8%) | 21 (24.1%) | 0.29 |
| *ACVR1B* | 16 (3.1%) | 14 (3.3%) | 2 (2.3%) | 1.00 |
| *ACVR2A* | 49 (9.5%) | 44 (10.3%) | 5 (5.7%) | 0.23 |
| *SMAD2* | 21 (4.1%) | 18 (4.2%) | 3 (3.4%) | 1.00 |
| *SMAD3* | 16 (3.1%) | 15 (3.5%) | 1 (1.1%) | 0.49 |
| *SMAD4* | 74 (14.3%) | 65 (15.2%) | 9 (10.3%) | 0.24 |
| *TGFBR1* | 5 (1.0%) | 4 (0.9%) | 1 (1.1%) | 1.00 |
| *TGFBR2* | 23 (4.5%) | 21 (4.9%) | 2 (2.3%) | 0.40 |

**Table S2. Mutation rate of critical pathways according to histology**

| Gene | Total  (*N* = 87) | Non-MAC  (*N* = 78) | MAC  (*N* = 9) | *P-*Value |
| --- | --- | --- | --- | --- |
| WNT Pathway | 69 (67.8%) | 64 (82.1%) | 5 (55.6%) | 0.084 |
| P53 pathway | 60 (69.0%) | 56 (71.8) | 4 (44.4%) | 0.13 |
| RTK-RAS pathway | 57 (65.5%) | 49 (92.8%) | 8 (88.9%) | 0.16 |
| PI3K pathway | 19 (21.8%) | 15 (19.2%) | 4 (44.4%) | 0.10 |
| TGF-β pathway | 21 (24.1%) | 13 (16.7%) | 8 (88.9%) | < 0.001 |

Abbreviations: MAC, mucinous adenocarcinoma.

**Table S3. Survival after recurrence according to each gene mutation. (Genes with mutation rate over 5%)**

| Gene | Mutation rate | Wild type  Median SAR  (months) | Mutation  Median SAR  (months) | *P-*Value |
| --- | --- | --- | --- | --- |
| WNT Pathway |  |  |  |  |
| *AMER1* | 8 (9.2%) | 38.2 | 23.6 | 0.53 |
| *APC* | 56 (64.4%) | 28.1 | 45.6 | 0.070 |
| *ARID1A* | 7 (8.0%) | 36.7 | 55.4 | 0.49 |
| *AXIN2* | 5 (5.7%) | - | - | - |
| *CTNNB1* | 1 (1.1%) | - | - | - |
| *DKK1* | 0 (0.0%) | - | - | - |
| *DKK2* | 0 (0.0%) | - | - | - |
| *DKK3* | 0 (0.0%) | - | - | - |
| *DKK4* | 1 (1.1%) | - | - | - |
| *FBXW7* | 14 (16.1%) | 37.8 | 44.4 | 0.58 |
| *FZD10* | 2 (2.3%) | - | - | - |
| *LRP5* | 3 (3.4%) | - | - | - |
| *SOX9* | 6 (6.9%) | 38.2 | 37.8 | 0.72 |
| *TCF7L2* | 6 (6.9%) | 38.7 | 31.7 | 0.59 |
| P53 pathway |  |  |  |  |
| *ATM* | 5 (5.7%) | 38.2 | 60.0 | 0.45 |
| *TP53* | 57 (65.5%) | 36.7 | 38.7 | 0.62 |
| RTK-RAS pathway | |  |  |  |
| *BRAF* | 8 (9.2%) | 38.2 | 19.8 | 0.42 |
| *EGFR* | 2 (2.3%) | - | - | - |
| *ERBB2* | 5 (5.7%) | 38.2 | 11.8 | 0.70 |
| *ERBB3* | 4 (4.6%) | - | - | - |
| *ERBB4* | 3 (3.4%) | - | - | - |
| *HRAS* | 0 (0.0%) | - | - | - |
| *KRAS* | 42 (48.3%) | 37.8 | 44.4 | 0.71 |
| *NRAS* | 3 (3.4%) | - | - | - |
| PI3K pathway | |  |  |  |
| *IGF1R* | 1 (1.1%) | - | - | - |
| *INS-IGF2* | 4 (4.6%) | - | - | - |
| *IRS2* | 0 (0.0%) | - | - | - |
| *MTOR* | 2 (2.3%) | - | - | - |
| *PDGFRA* | 1 (1.1%) | - | - | - |
| *PIK3CA* | 8 (9.2%) | 37.8 | 38.2 | 0.74 |
| *PIK3R1* | 2 (2.3%) | - | - | - |
| *PTEN* | 1 (1.1%) | - | - | - |
| *SRC* | 2 (2.3%) | - | - | - |
| TGF-β pathway | |  |  |  |
| *ACVR1B* | 2 (2.3%) | - | - | - |
| *ACVR2A* | 5 (5.7%) | 38.2 | 36.7 | 0.69 |
| *SMAD2* | 3 (3.4%) | - | - | - |
| *SMAD3* | 1 (1.1%) | - | - | - |
| *SMAD4* | 9 (10.3%) | 38.7 | 17.6 | 0.031 |
| *TGFBR1* | 1 (1.1%) | - | - | - |
| *TGFBR2* | 2 (2.3%) | - | - | - |

**Table S4. Detailed profile of TGF-β pathway mutation**

| **GENE** | **MUTATION_TYPE** | **AA_CHANGE** | **SEQ_CHANGE** | **PROVEAN** | **SIFT** | **PolyPhen-2** | **OncoKB** |
| --- | --- | --- | --- | --- | --- | --- | --- |
| ACVR1B | nonsynonymous SNV | p.G407R | c.G1219A | Deleterious | Damaging | Probably damaging | NA |
| ACVR1B | nonsynonymous SNV | p.R201H | c.G602A | Deleterious | Damaging | Probably damaging | NA |
| ACVR1B | stopgain SNV | p.R444X | c.C1330T | NA | NA (stop gain) | NA | NA |
| ACVR2A | nonsynonymous SNV | p.F61L | c.T183G | Deleterious | Damaging | Possibly damaging | NA |
| ACVR2A | frameshift deletion | p.K437fs | c.1310delA | NA | NA | NA | NA |
| ACVR2A | frameshift deletion | p.K437fs | c.1310delA | NA | NA | NA | NA |
| ACVR2A | frameshift deletion | p.K437fs | c.1310delA | NA | NA | NA | NA |
| ACVR2A | frameshift deletion | p.P182fs | c.545delC | NA | NA | NA | NA |
| SMAD2 | stopgain SNV | p.C433X | c.C1299A | NA | NA | NA | NA |
| SMAD2 | nonsynonymous SNV | p.F272S | c.T815C | Deleterious | Damaging | Possibly damaging | NA |
| SMAD2 | nonsynonymous SNV | p.T213P | c.A637C | Neutral | Tolerated | Benign | NA |
| SMAD3 | nonsynonymous SNV | p.R73C | c.C217T | Deleterious | Damaging | Possibly damaging | NA |
| SMAD4 | nonframeshift deletion | p.536_538del | c.1608_1613del | Deleterious | NA | NA | NA |
| SMAD4 | nonsynonymous SNV | p.P130S | c.C388T | Deleterious | Damaging | Probably damaging | Loss of function |
| SMAD4 | nonsynonymous SNV | p.P356L | c.C1067T | Deleterious | Damaging | Probably damaging | NA |
| SMAD4 | stopgain SNV | p.Q256X | c.C766T | NA | NA | NA | NA |
| SMAD4 | nonsynonymous SNV | p.R361C | c.C1081T | Deleterious | Damaging | Probably damaging | Loss of function |
| SMAD4 | nonsynonymous SNV | p.R361C | c.C1081T | Deleterious | Damaging | Probably damaging | Loss of function |
| SMAD4 | nonsynonymous SNV | p.R361H | c.G1082A | Deleterious | Damaging | Probably damaging | R361C loss of function |
| SMAD4 | nonsynonymous SNV | p.R361H | c.G1082A | Deleterious | Damaging | Probably damaging | R361C loss of function |
| SMAD4 | stopgain SNV | p.R445X | c.C1333T | NA | NA | NA | NA |
| TGFBR1 | nonsynonymous SNV | p.G137V | c.G410T | Deleterious | Damaging | Probably damaging | NA |
| TGFBR1 | stopgain SNV | p.W143X | c.G429A | NA | NA | NA | NA |
| TGFBR2 | nonframeshift deletion | p.I98del | c.293_295del | Deleterious | NA | NA | NA |
| TGFBR2 | nonsynonymous SNV | p.S46R | c.A136C | Neutral | Damaging | NA | NA |

Abbreviations: NA, not available
