# Supplementary figures and images for: Association of pathway mutation with survival after recurrence in colorectal cancer patients treated with adjuvant fluoropyrimidine and oxaliplatin chemotherapy
Source: BMC Cancer. 2019 May 6;19:421. doi: 10.1186/s12885-019-5650-0 (PMC6501409; doi:10.1186/s12885-019-5650-0)

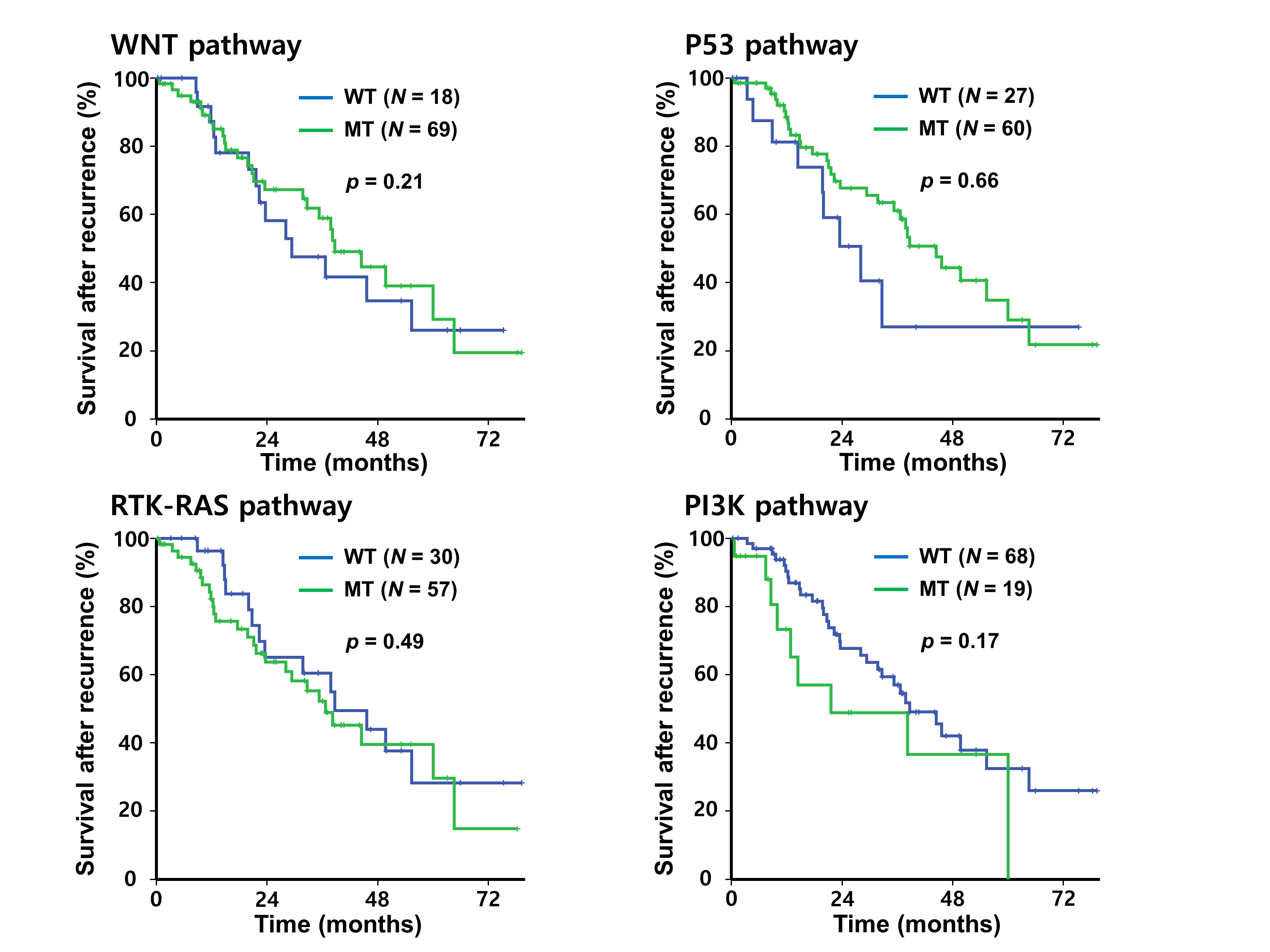

Supplement: Supplementary file 2 — Figure S1. Pathway mutations and survival after recurrence. Mutation in pathways other than TGF-β were not associated with survival after recurrence. (TIF 311 kb) [file 12885_2019_5650_MOESM2_ESM.tif]
